# Genetic interaction of *P2X_7_* receptor and *VEGFR-2* polymorphisms identifies a favorable prognostic profile in prostate cancer patients

**DOI:** 10.18632/oncotarget.4926

**Published:** 2015-08-21

**Authors:** Anna Solini, Vittorio Simeon, Lisa Derosa, Paola Orlandi, Chiara Rossi, Andrea Fontana, Luca Galli, Teresa Di Desidero, Anna Fioravanti, Sara Lucchesi, Luigi Coltelli, Laura Ginocchi, Giacomo Allegrini, Romano Danesi, Alfredo Falcone, Guido Bocci

**Affiliations:** ^1^ Department of Clinical and Experimental Medicine, University of Pisa, Pisa, Italy; ^2^ Laboratory of Pre-clinical and Translational Research, IRCCS - CROB Referral Cancer Center of Basilicata, Rionero in Vulture, Potenza, Italy; ^3^ Oncology Unit 2, University Hospital of Pisa, Pisa, Italy; ^4^ Division of Medical Oncology, Pontedera Hospital, Azienda USL of Pisa, Pontedera, Italy

**Keywords:** survival dimensionality reduction analysis, P2X_7_ receptor, VEGFR-2, polymorphisms, prostate cancer

## Abstract

VEGFR-2 and P2X_7_ receptor (P2X_7_R) have been described to stimulate the angiogenesis and inflammatory processes of prostate cancer. The present study has been performed to investigate the genetic interactions among *VEGFR-2* and *P2X_7_R* SNPs and their correlation with overall survival (OS) in a population of metastatic prostate cancer patients. Analyses were performed on germline DNA obtained from blood samples and SNPs were investigated by real-time PCR technique. The survival dimensionality reduction (SDR) methodology was applied to investigate the genetic interaction between SNPs. One hundred patients were enrolled. The SDR software provided two genetic interaction profiles consisting of the combination between specific *VEGFR-2* (rs2071559, rs11133360) and *P2X_7_R* (rs3751143, rs208294) genotypes. The median OS was 126 months (95% CI, 115.94–152.96) and 65.65 months (95% CI, 52.95–76.53) for the favorable and the unfavorable genetic profile, respectively (*p* < 0.0001). The genetic statistical interaction between *VEGFR-2* (rs2071559, rs11133360) and *P2X_7_R* (rs3751143, rs208294) genotypes may identify a population of prostate cancer patients with a better prognosis.

## INTRODUCTION

The prostate cancer progression has been related to many factors such as inflammation [1] and the so-called angiogenic switch that implies enhanced angiogenesis, characterized by high vascular endothelial growth factor (VEGF) and VEGF receptor (VEGFR) levels [2]. Although significance of inflammation in cancer progression is still a debated issue, some evidences suggest pathways directed to connection between inflammation and cancer evolution [3]. Indeed, the proliferative inflammatory atrophy has been proposed as possible precursor of prostatic intraepithelial neoplasm. Since inflammatory microenvironment releases growth factors (such as VEGF) and cytokines, proangiogenic factors may influence the activation of the vascular endothelial cells and signal transduction in these cells [3]. Angiogenesis is an important step in the development of cancer and is necessary for primary tumor growth, invasiveness, and metastasis. This neovessel formation is stimulated by different proangiogenic factors secreted by cells pertaining to the tumor itself and to its microenvironment [4]. Kinase insert domain receptor (KDR or VEGFR-2) is the principal receptor that promotes the pro-angiogenic action of vascular endothelial growth factor and is recognized as the main target of anti-angiogenic therapies.

The extracellular signalling molecule ATP (eATP) has been shown to mediate various biological functions including synaptic neurotransmission, nociception, smooth muscle contraction, and endocrine secretion [5, 6]. eATP acts via specific P2 receptors, ion-gated channels classified into ionotropic (P2X) and metabotropic (P2Y) subtypes. In the last fifteen years, attention has been paid to the complex relationship between purinergic signal and cancer [5, 7]. Among the various ATP-sensitive receptors, the P2X_7_ receptor (P2X_7_R) has attracted our attention as potential mediator of the cellular processes described above, especially in prostatic carcinoma, where an increased P2X_7_R expression has been reported, irrespective of Gleason grade or patient age [8]. Moreover, Ravenna and colleagues have suggested that an up-regulation of P2X_7_R in bioptic specimens of prostate cancer might trigger the generation of other pro-inflammatory molecules through NF-kB activation [9].

The gene encoding for P2X_7_R, located at chromosome position 12q24, is highly polymorphic. Several loss-of-function polymorphisms may alter the normal trafficking and activity of the receptor [10, 11]. The *VEGFR-2* gene is located on chromosome 4q11-q12. Several single-nucleotide polymorphisms (SNPs) have been described in the *VEGFR-2* gene, some of which able to increase/decrease gene expression itself, circulating levels of soluble VEGFR-2 and the VEGF binding efficiency to the receptor [12]. Many authors have pointed out the unlikeliness that just a SNP can predict the prognosis or the therapeutic response to drugs, mainly due to the complexity of the involved biological systems [4, 13, 14]. Therefore, the prognosis of advanced prostate cancer could depend on many factors, likely interacting with a complex genetic background. Indeed, the effect of a SNP on its corresponding gene may be the result of an interaction between the polymorphisms of other functionally linked genes. This phenomenon is defined as epistasis [15, 16]. Gene–gene interactions are an essential part of gene regulation, signal transduction, biochemical networks, and homeostatic, developmental, and physiological pathways. Indeed, the epistasis phenomenon involves DNA sequence variations, biomolecules and their physical interactions giving rise to a phenotype at a particular time point [17]. Therefore, the current approach of correlating the prostate cancer prognosis to a single SNP may be replaced by a genetic analysis of the interaction between SNPs. The aim of this new approach should be to unveil a genetic profile with a reliable prognostic value. Beretta and colleagues have created and validated a methodology called survival dimensionality reduction (SDR), an innovative approach to detect epistasis in presence of right-censored data, to identify a genetic profile with the ability to predict a better survival of patients [18].

Based on these hypotheses, we conducted a retrospective study to assess the ability of SDR methodology to identify a genetic profile of *VEGFR-2* and *P2X_7_R* polymorphisms associated to the overall survival (OS) in an unselected population of prostate cancer patients.

## RESULTS

One-hundred patients with histological diagnosis of prostate adenocarcinoma were enrolled into the study (Table 1). Ninety-three patients were treated with chemotherapy (45 patients received more than 2 lines of chemotherapy) such as docetaxel, estramustine, mitoxantrone, cyclophosphamide, and vinorelbine. Radiotherapy was administered in 6 patients, whereas 95 patients received androgen deprivation therapy soon after the prostate cancer diagnosis or at the time of the metastatic disease. Thirty-four patients underwent surgery.

**Table 1:** Patients Characteristics (*n* = 100)

		N°		%
**Age**				
	Median		70.5	
	Range		48–91	
**ECOG Performance Status**				
	0	71		71
	1	22		22
	2	5		5
	Not available	2		2
**Metastatic Disease at the Diagnosis**				
	Yes	44		44
	No	51		51
	Not available	5		5
**Sites of Metastatic Disease at the Diagnosis**				
	Bone and Nodes	7		7
	Bone	31		31
	Nodes	6		6
	Not available	5		5
**Hormonal Manipulations**				
	Yes	95		95
	No	5		5
**Surgery**		34		34
**Radiotherapy**		6		6
**Chemotherapy**				
	Yes	93		93
	No	5		5
	Not available	2		2
**Number of Lines of Chemotherapy**				
	1	24		24
	≥ 2	67		67
	Not available	2		2
**Serum PSA at the Diagnosis**				
	Median (ng/ml)		99	
	Range		2.36–5307	
**Gleason Score at the Diagnosis**				
	<7	12		12
	≥7	56		56
	Not available	32		32

In Table 2 the associations of clinical and pathological characteristics with OS are reported. The univariate Cox regression analysis confirmed the expected role of the Gleason score, the ECOG PS and the number of metastatic sites in determining the prognosis of this group of patients.

**Table 2:** Association between clinical and pathological characteristics with overall survival in the whole study cohort

Variables	HR	*p*	95%CI
Age	0.99	0.88	0.96 – 1.03
ECOG PS	1.89	0.009	1.17 – 3.06
PSA at the diagnosis	1.018	0.366	0.98 – 1.06
Gleason Score	3.59	0.019	1.23 – 10.44
MTS sites at the diagnosis	1.51	0.061	0.98 – 2.32
Therapy	1.39	0.184	0.85 – 2.27

ECOG PS, Eastern Cooperative Oncology Group performance status; HR, hazard ratio; CI, confidence interval; MTS, metastases; PSA, prostate-specific antigen. ECOG PS and MTS were analysed as continuous variable. Gleason Score represents the risk difference between patients with <7 (reference) and ≥7. Therapy represents patients who received chemotherapy (none, 1 and ≥ 2 lines) and hormonal treatment between diagnosis and metastasis period.

Details about genotypes and allele frequencies of all SNPs in the studied population are reported in Table 3; all the SNPs were in HWE. None of the single SNPs showed a statistically significant association in univariate Cox regression analysis, either under an additive, dominant or recessive model as illustrated in Table 4, with the only exception of *VEGFR-2* rs2305948 that reaches the significance threshold in the dominant model. However, this statistically significant association is certainly a false-positive result due to the large confidence interval and the occurrence of just one TT case. Furthermore, the haplotype analysis of the two genetic regions did not reveal any significant association in univariate Cox regression model and Kaplan Meier curves (Supplementary Figure 1).

**Table 3:** Polymorphisms, genotypes, allele frequencies and Hardy-Weinberg Equilibrium (HWE)

*ID*	*Gene*	*TaqMan SNPgenotyping assays*	*genotype*	*N*	*allele*	*N*	*%*	*HWE *p*-value*
rs2071559	*VEGFR-2*	C__15869271_10	AA	29	A	107	0.54	0.8435
			AG	49	G	93	0.46	
			GG	22				
rs2305948	*VEGFR-2*	C__22271999_20	CC	89	C	188	0.94	0.2956
			CT	10	T	12	0.06	
			TT	1				
rs1870377	*VEGFR-2*	C__11895315_20	TT	72	T	169	0.84	0.7
			AT	25	A	31	0.16	
			AA	3				
rs11133360	*VEGFR-2*	C__26111278_10	TT	30	T	110	0.55	1
			CT	50	C	90	0.45	
			CC	20				
rs3751143	*P2X_7_R*	C__27495274_10	AA	60	A	150	0.75	0.0589
			AC	30	C	50	0.25	
			CC	10				
rs208294	*P2X_7_R*	C___3019032_1_	CC	29	C	106	0.53	0.6926
			CT	48	T	94	0.47	
			TT	23				

**Table 4:** Association between each polymorphism and overall survival (univariate Cox regression model) A *p* value < 0.0083 was defined as statistically significant (Bonferroni's correction). *, the statistically significant association is a false-positive result due to the large confidence interval and the occurrence of only one TT case

		Additive model					Dominant model					Recessive model				
*ID*	*Gene*	*genotype*	*N*	*HR*	*p*	*95%CI*	*genotype*	*N*	*HR*	*p*	*95%CI*	*genotype*	*N*	*HR*	*P*	*95%CI*
rs 2071559	*VEGFR-2*	AA	29	1	-	-						AA	29	1	-	-
		AG	49	1.06	0.85	0.56 – 1.99	AA/AG	78	1	-	-					
		GG	22	0.97	0.94	0.46 – 2.04	GG	22	0.93	0.83	0.49 – 1.75	AG/GG	71	1.03	0.91	0.57 – 1.86
rs 2305948	*VEGFR-2*	CC	89	1	–	–						CC	89	1	–	–
		CT	10	0.7	0.59	0.47 – 3.7	CC/CT	99	1	–	–					
		TT	1	22.3	0.006*	2.5 – 200.5	TT	1	21.7	0.006*	2.42 – 194.1	CT/TT	11	1.63	0.3	0.64 – 4.1
rs 1870377	*VEGFR-2*	TT	72	1	–	–						TT	72	1	–	–
		AT	25	1.34	0.37	0.7 – 2.55	TT/AT	97	1	–	–					
		AA	3	1.64	0.49	0.4 – 6.9	AA	3	1.53	0.56	0.37 – 6.35	AT/AA	28	1.37	0.3	0.74 – 2.53
rs 11133360	*VEGFR-2*	TT	30	1	–	–						TT	30	1	–	–
		CT	50	0.72	0.3	0.38 – 1.35	TT/CT	80	1	–	–					
		CC	20	0.9	0.78	0.44 – 1.85	CC	20	1.12	0.69	0.62 – 2.04	CT/CC	70	0.77	0.4	0.43 – 1.4
rs 3751143	*P2X_7_R*	AA	60	1	–	–						AA	60	1	–	–
		AC	30	0.68	0.19	0.38 – 1.22	AA/AC	90	1	–	–					
		CC	10	0.66	0.48	0.2 – 2.15	CC	10	0.75	0.64	0.23 – 2.4	AC/CC	40	0.68	0.16	0.39 – 1.17
rs 208294	*P2X_7_R*	CC	29	1	–	–						CC	29	1	–	–
		CT	48	1.16	0.64	0.62 – 2.19	CC/CT	77	1	–	–					
		TT	23	1.1	0,8	0.49 – 2.49	TT	23	1.001	0.99	0.5 – 1.99	CT/TT	71	1.14	0.65	0.62 – 2.1

**Figure 1:**
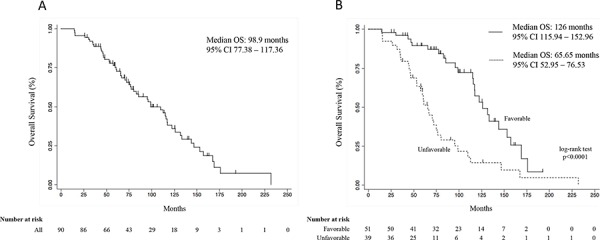
Overall survival curve of all patients (A), and overall survival curves according to the favorable and unfavorable genetic profiles (B). Survival curves were calculated by the Kaplan Meier method *CI, confidence interval*.

The SDR analysis revealed a genetic interaction profile, consisting of the combination between specific *VEGFR-2* (rs2071559, rs11133360) and *P2X_7_R* (rs3751143, rs208294) genotypes, significantly correlated with OS. Table SDR model (Table 5) shows the full analysis conducted by the SDR algorithm in our datasets. Cross-validation prevented over-fitting as the minimum test-IBS (0.1689) was observed for the interaction model, which was therefore chosen as the best epistatic model (i.e. the 4-way SDR model). This model was highly significant at the 0.05 threshold after 1000-fold permutation testing (*p* < 0.001). In particular, two genetic profiles were identified in patients, as reported in the genetic model table (Table 6). The favorable genetic profile was associated with a greater OS benefit whereas the unfavorable one with a lower OS in the enrolled patients, as shown in Figure 1. Indeed, after a median follow-up of 74+ months (range 14.6 – 231.7+ months), median OS of the enrolled patients was 98.9 months (Figure 1A; 95% CI, 77.38–117.36). Notably, the median OS for the favorable genetic profile was 126 months (Figure 1B; 95% CI, 115.94–152.96 months) *vs.* the 65.65 months of the unfavorable genetic profile (95%CI, 52.95–76.53 months; *p* < 0.0001, log-rank test; Figure 1B). Moreover, the Supplementary Figure 2 also shows the median OS of favorable and unfavorable profiles analyzed by the 3-way SDR analysis. The Cox proportional hazards model, performed to assess the adjusted hazard ratio for the OS of the favorable genetic profile, showed a value of 0.29 (95%CI, 0.15–0.56; *p* = 0.0001; Table 7). Supplementary Table 1 shows the same multivariate Cox regression model including also the variables “therapy” and “metastases sites at the diagnosis” that resulted not significant at the univariate analysis, confirming the lack of influence, in our model, of these two variables on OS. Of note, the probability of an estimated 2-year survival rate was 98% (95% CI, 87–99) in the favorable genetic profile and 92% (95% CI, 78–97) in the unfavorable genetic profile; the estimated 3-year survival was 96% (95% CI, 85–99) and 82% (95% CI, 66–91), respectively. Supplementary Table 2 shows the patient's characteristics of both favorable and unfavorable groups. No differences were noted with the exception of the number of metastases sites at the diagnosis. Anyway, this variable was used in multivariate Cox regression to obtain an adjusted model (see Supplementary Table 1). However, this analysis confirms that the MTS sites at diagnosis, at least in our model, did not influence significantly the difference in OS between the two groups.

**Table 5:** Survival dimensionality reduction (SDR) model for the survival analysis of dataset

		IBS		
*n*-way	SNPs (genes) in each dimension	Training	Testing	*p*
1	rs3751143 (*P2X_7_R*)	0.1876	0.1877	
2	rs1870377 (*VEGFR-2*), rs3751143 (*P2X_7_R*)	0.1846	0.1852	
3	rs11133360 (*VEGFR-2*), rs3751143 (*P2X_7_R*), rs208294 (*P2X_7_R*)	0.1741	0.1784	<0.001
**4**	**rs2071559 (*VEGFR-2*), rs11133360 (*VEGFR-2*), rs3751143 (*P2X_7_R*), rs208294 (*P2X_7_R*)**	**0.1638**	**0.1689**	**<0.001**

Selection of the best combination of attributes by the SDR method. The model with the minimum testing Integrated Brier Score (IBS) value in the cross-validated testing sets is indicated in boldface type. *P* values associated with the log-rank test statistic calculated by the 1000-fold permutation test. SNP, single nucleotide polymorphism.

**Table 6:** Results of the genetic interaction analysis to translate the genotype combinations of the *VEGFR-2* (rs2071559, rs11133360) and *P2X_7_R* (rs3751143, rs208294) genotypes into favorable or unfavorable genetic profiles for overall survival

Favorable genetic profile				Unfavorable genetic profile			
rs2071559	rs11133360	rs3751143	rs208294	rs2071559	rs11133360	rs3751143	rs208294
AA	CC	AA	CC	AA	CC	AA	CT
AA	CC	AA	TT	AA	CC	AC	CT
AA	CC	CC	CT	AA	CT	AA	TT
AA	CT	AA	CC	AA	CT	AC	CC
AA	CT	AA	CT	AG	CC	AA	CC
AA	CT	AC	CT	AG	CC	AA	CT
AA	CT	AC	TT	AG	CT	AA	CT
AA	TT	AA	CC	AG	CT	AC	TT
AA	TT	AC	CT	AG	TT	AA	CC
AA	TT	AC	TT	AG	TT	AC	CT
AG	CC	AC	CT	AG	TT	AC	TT
AG	CC	AC	TT	GG	CC	CC	TT
AG	CT	AA	CC	GG	CT	AA	TT
AG	CT	AA	TT	GG	CT	AC	CC
AG	CT	AC	CC	GG	TT	AA	CC
AG	CT	AC	CT	GG	TT	AA	CT
AG	TT	AA	CT				
AG	TT	CC	CT				
AG	TT	CC	TT				
GG	CC	AC	CT				
GG	CT	AA	CC				
GG	CT	AA	CT				
GG	CT	CC	CC				
GG	CT	CC	TT				
GG	TT	AC	CT				

**Table 7:** Multivariate Cox regression model

*Variables*	*HR*	*p*	*95%CI*
Age	0.93	0.008	0.88 – 0.98
ECOG PS	1.40	0.34	0.7 – 2.8
Gleason Score	5.18	0.005	1.65 – 16.2
*Genetic profile*			
Unfavorable	1	–	–
Favorable	0.29	0.0001	0.15 – 0.56

ECOG PS, Eastern Cooperative Oncology Group performance status; HR, hazard ratio; CI, confidence interval; ECOG PS was analysed as continuous variable. Gleason Score represents the risk difference between patients with <7 (reference) and ≥7.

## DISCUSSION

Prostate carcinoma is still a leading cause of morbidity and mortality in developed countries, and the expected number of cancer cases in the next 15 years will increase by > 20% [19]. Localized cancers are usually treated with radical prostatectomy or radiation, while for more advanced cancers, either recurred or metastasized, the gold standard treatment is androgen ablation therapy, followed by the administration of chemotherapeutic drugs (e.g. docetaxel) [20].

The identification of biomarkers able to identify patients with the best prognosis is urgently needed to better define the proper therapeutic approach in the management of this disease. In the present analysis, we have applied the SDR methodology for identifying a genetic profile consisting of the combination between specific *VEGFR-2* (rs2071559, rs11133360) and *P2X_7_R* (rs3751143, rs208294) genotypes associated with a greater OS as compared to that observed in the whole study population, in an unselected population of patients with prostate cancer. Among several loss-of-function SNPs of the P2X_7_R gene that may alter the normal trafficking [21] and activity of the receptor, the rs3751143 polymorphism has been associated to the familial form of chronic lymphocytic leukemia [22] and to the follicular variant of papillary thyroid cancer [23]. On the other hand, the polymorphism rs208294, leading the substitution of histidine for tyrosine at position 155 (H155Y) localized in P2X_7_R ectodomain, causes a gain of function of the receptor and it has been linked with human chronic lymphocytic leukemia lymphocytes [24]. Tumor formed by K562 cells transfected for a new mutation (A^559^-to-G substitution) showed a proliferative advantage and higher expression of VEGF and MCP1 [25], and haplotypes of the *P2X_7_R* containing the rs1718119 polymorphism have been linked to an enhanced IL-1β secretion [10]. The association between the *VEGFR-2* SNPs and susceptibility or prognosis in several different types of cancers, such as renal cell carcinoma, colorectal cancer, hepatocellular carcinoma and glioblastoma, has been studied [26–29]. The rs2071559 G > A SNP has been reported to alter the structure of the binding site in *VEGFR-2* gene promoter region for transcriptional factor E2F, with a consequent decrease of receptor expression, whereas the exonic polymorphisms rs1870377 and rs2305948 (in exon 11 and exon 7, respectively) resulted in a significant reduction of the VEGF binding efficiency to VEGFR-2 [12]. Moreover, Glubb and colleagues found that the rs1870377 T > A increases VEGFR-2 protein phosphorylation, thus resulting in an increased microvessel density in lung cancer tumor tissues [30].

In our study we firstly associated each individual SNP with OS, but no association between each polymorphism and OS was found with the only exception of *VEGFR-2* rs2305948, a false-positive result due to the large confidence interval and the occurrence of just one TT case. As demonstrated by the epistasis phenomenon, the effects of a single SNP can disseminate through numerous gene–gene interactions, determining multiple phenotypes; thus, we think that the current approach of correlating the prognosis to just one SNP should be reconsidered and replaced by a genetic analysis of the interaction among SNPs. For these reasons, we decided to apply the more complex SDR methodology to investigate the interaction between germline SNPs, in order to identify a possible genetic profile associated with the greater OS probability in this unselected population of patients carrying prostate cancer (Table 1 and 5). The analyses conducted in this population revealed a genetic interaction profile, consisting of the combination between specific *VEGFR-2* (rs2071559, rs11133360) and *P2X_7_R* (rs3751143, rs208294) genotypes, associated with OS. Particularly, two genetic profiles were identified in patients, as reported in Table 6. The first column was associated with a greater OS benefit and the second one with a lower OS, respectively. Because of the connections within biological pathways, the effects of a single mutation can extend through thousands of gene–gene interactions, resulting in multiple phenotypes [31]. In our study we demonstrated, through the SDR methodology, a never previously shown statistically significant interaction between *P2X_7_R* and *VEGFR-2* gene SNPs that potentially relates to prognosis of prostate cancer patients.

Genetic information impacts phenotype through a hierarchy of proteins that are involved in biological processes ranging from transcription to physiological homeostasis. Physical interactions among proteins and other biomolecules and their impact on phenotype are the main components of biological epistasis [32]. Biological epistasis occurs at the level of the single patient and involves DNA sequence variations, biomolecules and their physical interactions determining a particular phenotype for that single individual. Instead, statistical epistasis is a population event that is made possible by interindividual variability in genotypes, biomolecules and in their physical interactions [32]. Thus, the differences in biological epistasis among individuals in a population give rise to statistical epistasis [32]. Evidently, making both hypotheses or conclusions about biological function and causation from statistical epistasis results in patients will always balk in a challenge, unless relevant molecular information has been also acquired [33]. However, SDR or multifactor dimensionality reduction (MDR) methodology [34] has been applied to detect gene-gene interactions for several clinical phenotypes, and it may provide means to find new hypotheses for further testing about epistatic interactions in genetic data also in prostate cancer. As an example, the interaction between VEGF 2482T and VEGFR IVS6 + 54 loci suggests that the inheritance of VEGF and VEGFR IVS6 + 54 sequence variants may jointly modify prostate cancer susceptibility through their influence on angiogenesis [35]. Whilst it is difficult to explain the biological meaning of our statistical epistatic data, it should be noted that several data support a possible link between *P2X_7_R* and VEGFR-2 in tumor physiopathology. Although at a first glance the two genes, and, consequently, the two receptors have completely different activities and signaling pathways, it is not surprising that P2X_7_R and VEGFR-2 share more than a simple biological connection. In fact, it has been demonstrated that the modulation of angiogenesis exerted by eATP through endothelial P2Y receptors can stimulate angiogenic properties such as endothelial cell migration and vascular permeability [36, 37]; moreover activated P2Y_2_ receptors can transactivate VEGFR-2, suggesting a direct link between extracellular nucleotide regulation and growth factor signaling [38]. Interestingly, Wei and colleagues [39] demonstrated that the chronic exposure of C6 glioma cells to 2′,3′-(benzoyl-4-benzoyl)-ATP (BzATP), a selective P2X_7_R agonist, enhanced the expression of pro-inflammatory and angiogenic factors, including IL-8 and VEGF, suggesting a link between the receptor activation, inflammation, increased angiogenesis and tumor cell migration. Furthermore, P2X_7_R-expressing tumors are characterized by increased proliferation, reduced apoptosis, and a more developed vascular network that control tumors and by elevated secreted amounts of VEGF [40]. The growth and neoangiogenesis of P2X_7_R-expressing tumors was blocked by intratumoral injection of the VEGF-blocking antibody bevacizumab, pharmacologic P2X_7_R blockade, or *P2X_7_R* silencing *in vivo* [40]. Recently, Amoroso and colleagues showed that P2X_7_R down-modulation caused a reduction in HIF1alpha levels and VEGF secretion with a decreased vessel formation in a neuroblastoma model [41]. Based on these premises, it is conceivable to hypothesize that, in patients carrying the more unfavorable genetic profile, the pro-inflammatory activity and the tumor angiogenesis is sustained by the gene-gene interaction of both receptors, with a graduality due to the various combinations of genotypes, resulting in a worse prognosis. Such angiogenic phenotype could be due to an increase of the VEGF production attributable to an enhanced P2X_7_R activity, which may in turn activate an upregulated VEGFR-2. Unfortunately, the phenotypes corresponding to the *VEGFR-2* rs11133360 genotypes are still unknown [42]. Therefore, it might be plausible that the genetic background may be responsible, in part, for the prognosis in these metastatic prostate cancer patients. Conversely, in patients with a more favorable genetic profile, the microenvironment conditions due to the different genotype combinations may result in a reduction of the VEGF production and in the presence of fewer VEGFR-2, or of its lower activity, on tumor endothelial cells which are not capable to proliferate, migrate or survive.

Thus, despite the objective difficulties to demonstrate a biological mechanism for the statistically demonstrated gene-gene interaction, our original findings may provide a new reliable prognostic value and suggest previously unknown hypotheses for further testing also biological epistatic interactions. Although this study presents some strengths as the long observational follow-up of the patients, lasting almost 20 years, or the extreme novelty of the approach, however, final considerations are restricted by the exploratory nature of its retrospective design, the limited sample size with the potential risk of an over-estimation of the obtained results. Indeed, these results should be only considered as “hypothesis generating” and only a well-designed prospective clinical trial may eventually confirm the suggested innovative role of the genetic interaction profile between specific *VEGFR-2* (rs2071559, rs11133360) and *P2X_7_R* (rs3751143, rs208294) genotypes analyzed by SDR. However, understanding the reasons why the singular genotypes of both *VEGFR-2* rs2071559 and rs11133360 or *P2X_7_R* rs3751143 and rs208294 were not associated to the greater benefit in terms of OS in our study, in contrast to what reported when combined in the interaction analysis, remains a challenge.

The identification of good *versus* poor prognosis prostate cancer patients through non-invasive and reliable means is a largely unmet clinical need. The possible clinical implication of identifying a favorable and an unfavorable group through SDR analysis could reside on this characteristic. In fact, we could hypothesize that poor prognosis patients may benefit from novel treatment strategies from the very beginning, and perhaps should be considered earlier for clinical trials with innovative drugs rather than waiting until the failure of standard therapies. On the other hand, prostate cancer patients with overall good prognosis may opt for a less aggressive treatment among the available standard therapeutic options.

In conclusion, the SDR methodology has been applied in these unselected prostate cancer patients to investigate the role of an interaction between *VEGFR-2* and *P2X_7_R* gene polymorphisms in identifying a genetic profile associated with the greater probability of OS. The results have confirmed the relevance of SDR analyses, as already described by other authors, suggesting a more rational approach when SNPs are investigated as possible predictors of prognosis.

## MATERIALS AND METHODS

### Study population

The present multicenter, retrospective, genetic study was approved by the local ethics committee. Patients were informed of the investigational nature of the study and provided their written informed consent for collecting genetic data. One-hundred patients with age ≥ 18 years and histological diagnosis of prostate adenocarcinoma were enrolled (Table 1), and they were assessed for the present genetic study. Other inclusion criteria were PSA ≥ 10 ng/mL; Eastern Cooperative Oncology Group performance status (ECOG PS) of ≤ 2; adequate bone marrow function (leukocytes, ≥ 3000/mL; neutrophil count, ≥ 1500/mL; hemoglobin level, ≥ 10 g/dL; platelets, ≥ 100,000/mL); adequate liver function (total serum bilirubin level, < 1.5 mg/dl; aspartate aminotransferase and alanine aminotransferase, < 3-fold upper limit of normal); adequate renal function (serum creatinine level, < 1.5 mg/dL), and life expectancy ≥ 3 months. Exclusion criteria at baseline were acute cardiovascular disease (uncontrolled hypertension and arrhythmia; myocardial infarction within 2 years before enrolment; unstable angina; New York Heart Association, grade II or greater congestive heart failure), active infections, high risk of thromboembolic events without prophylactic treatments, untreated hemorrhagic gastric disease, or presence of brain metastases. Sites of metastatic disease were evaluated by radiological exams (radiography, X-ray computed tomography) and ultrasonography (Table 1).

At the enrollment, clinical evaluations included: patient's medical history; a physical exam with an assessment of weight, vital signs, and ECOG PS; electrocardiogram plus cardiovascular examinations; complete blood count; complete serum biochemistry (i.e., serum creatinine, fasting glucose, sodium, potassium, calcium, lactate dehydrogenase, aspartate aminotransferase, alanine aminotransferase, γGT, ALP, total bilirubin, PT, aPTT, fibrinogen, and D-dimer), PSA serum levels.

### SNP selection

The SNPs included in our study (Table 3) were selected on the basis of three main considerations: (i) thus far no genetic interaction analysis has been reported between the chosen SNPs and prognosis of prostate cancer, and possible gene-gene interactions could determine previously undescribed statistical epistatic effects; (ii) some of the SNPs have been significantly associated with risk or prognosis in other tumour types [23, 26, 43–47]; (iii) some SNPs have been associated with a modulation of the gene expression, or with impaired activity of the receptors [10–12, 48]. Although for some of chosen SNPs the phenotypic effects are still undefined or controversial, we decided to include them in our research because of the possibility that gene-gene interaction could produce an unexpected statistical epistatic effect.

### Genotyping analyses

Blood samples (3 ml) were collected in EDTA tubes and stored at −80°C. *P2X_7_R* and *VEGFR-2* genes and polymorphisms of Table 3 were chosen for the present analyses. Germline DNA extraction was performed using QIAamp DNA Blood Mini Kit (Qiagen, Valencia, CA, USA). Allelic discrimination of genes was performed using an ABI PRISM 7900 SDS (Applied Biosystems, Carlsbad, CA, USA) and with validated TaqMan^®^ SNP genotyping assays (Table 3; Applied Biosystems). PCR reactions were carried out according to the manufacturer's protocol. Genotyping was not performed until an adequate number of events (>80% on study population) was reported in terms of OS.

### Statistical analysis

All polymorphisms were analyzed for deviation from the Hardy-Weinberg Equilibrium (HWE), through comparison between observed allelic distributions with those expected from the HWE by on *χ*2 test. Linkage disequilibrium (LD) between markers in *VEGFR-2* (*n* = 4) and *P2X_7_R* (*n* = 2) was analysed using Haploview software package [49]. The association between each individual polymorphism and the most relevant clinical-pathological characteristics with overall survival (OS) was tested using a Cox proportional hazards model. OS were calculated from the date of the diagnosis to the date of death/lost to follow-up.

PHASE software v 2.1 [50] was used to perform haplotype analysis for *VEGFR-2* and *P2X_7_R* regions separately. Only common haplotypes with a frequency ≥ 5% were included in COX survival analysis. Most common haplotype was used as the reference and were provided hazard ratios (HR) for a given haplotype relative to reference haplotype.

The Survival Dimensionality Reduction (SDR) methodology was applied (using version 2.0a of SDR software available on http://sourceforge.net/projects/sdrproject/) to detect non-linear gene-gene interactions in presence of right-censored data in identifying biomarkers of prognosis in OS [18]. The best combination of SDR model was chosen based on the lowest testing Integrated Brier Score (IBS) value. Log-rank test statistic calculated by the 100-fold permutation test was used to evaluate association and model efficiency.

The genotype combination with the OS benefit and improvement was used for further analyses. The difference in OS between favorable genetic profiles and the unfavorable genetic profiles were assessed with the log-rank test and the Kaplan-Meier method to evaluate survival curves. A Cox proportional hazards model, with the possible genetic profiles and the clinical and pathological patient characteristics individually correlated with the OS, was used to calculate the adjusted hazards ratio (HR) and the 95% confidence interval (95% CI). In these analyses we used Bonferroni's correction and the *p* value < 0.0083 (0.05/6 SNPs = 0.0083) was accepted as statistically significant. The Kaplan–Meier and Cox proportional hazards analyses were performed using the STATA package version 11.0 (StataCorp).

## SUPPLEMENTARY MATERIALS FIGURES AND TABLES


