# Supplementary material for: Genetic interaction of P2X7 receptor and VEGFR-2 polymorphisms identifies a favorable prognostic profile in prostate cancer patients
Source: Oncotarget. 2015 Aug 21;6(30):28743–54. doi: 10.18632/oncotarget.4926 (PMC4745689; doi:10.18632/oncotarget.4926)
Supplement: Supplementary file 1 [file oncotarget-06-28743-s001.pdf]

SUPPLEMENTARY FIGURES AND TABLES

A

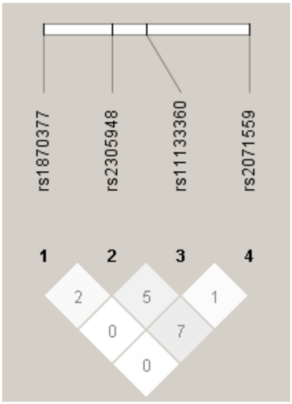

B

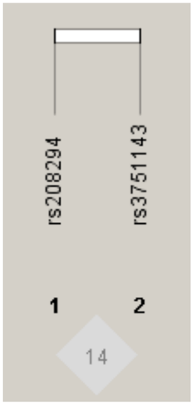

E

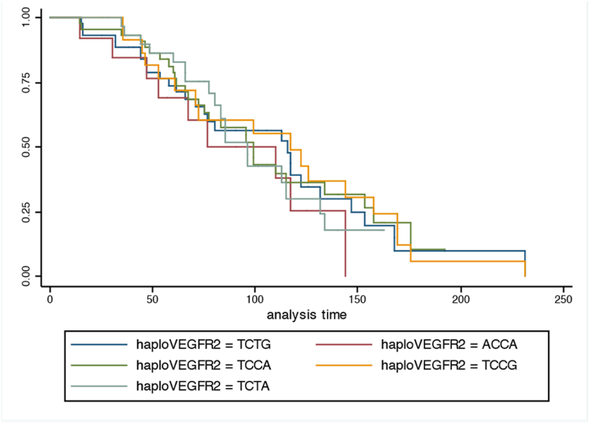

F

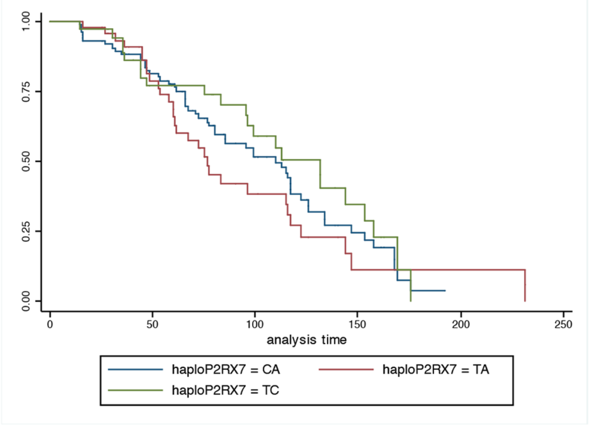

C

***VEGFR-2* haplotypes**	***%***
**TCTG**	**26.00**
ACCA	7.00
ACCG	0.50
ACTA	4.00
ACTG	1.50
ATTG	2.50
TCCA	25.00
TCCG	12.50
TCTA	17.50
TTTG	3.50

D

***$P2X_7R$* haplotypes**	***%***
**CA**	**49.00**
CC	4.00
TA	26.00
TC	21.00

G

Haplotype	HR	95% CI	*P*
TCTG	1	-	-
ACCA	1.47	0.69 - 3.16	0.32
TCCA	0.99	0.59 - 1.7	1.00
TCCG	0.95	0.52 - 1.73	0.86
TCTA	1.07	0.59 - 1.95	0.82

H

Haplotype	HR	95% CI	*P*
CA	1	-	-
TA	1.2	0.76 - 1.88	0.43
TC	0.85	0.52 - 1.4	0.51

Supplementary Figure S1: Haploview Linkage Disequilibrium Plots and Haplotype analysis in PHASE of *VEGFR-2* and *$P2X_7R$* gene region. Linkage disequilibrium (LD) was measured using r^2 in *VEGFR-2* **(A)**, and *$P2X_7R$* **(B)** gene region. Diamonds are white if $r^2 = 0$, varying shades of grey if $0 < r^2 < 1$, and are black if $r^2 = 1$. There is no linkage disequilibrium among these variants in both regions and none haplotype block was automatically called. An haplotype analysis was performed using PHASE. SNPs in *VEGFR-2* region were rs1870377 (A/T), rs2305948 (C/T), rs11133360 (C/T) and rs2071559 (A/G). SNPs in *$P2X_7R$* region were rs208294 (C/T), rs3751143 (A/C). Uncommon haplotypes (< 5%) were highlighted in red. Reference haplotype is marked with bold type. TCTG for *VEGFR-2* region **(C)** and CA for *$P2X_7R$* region **(D)**. None of the haplotype is significantly associated with survival in Kaplan Meier analysis for *VEGFR-2* **(E)** and *$P2X_7R$* **(F)** as well as in the univariate Cox model for *VEGFR-2* **(G)** and *$P2X_7R$* **(H)**, respectively.

A

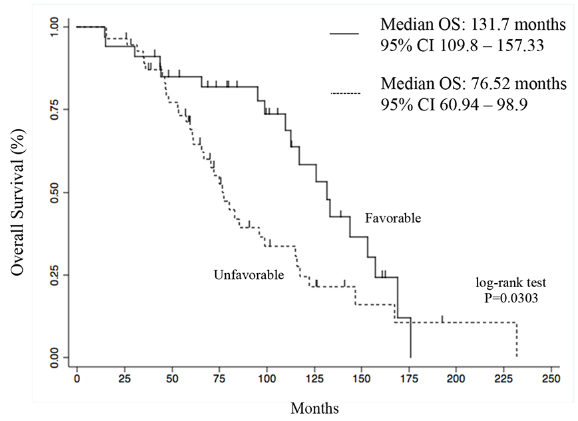

Number at risk

Favorable	34	32	27	23	17	11	6	1	0	0	0
Unfavorable	56	54	39	20	12	7	3	2	1	1	0

B

Favorable genetic profile			Unfavorable genetic profile		
rs11133360	*rs3751143*	*rs208294*	*rs11133360*	*rs3751143*	*rs208294*
CC	AA	TT	CC	AA	CC
CC	AC	CT	CC	AA	CT
CC	AC	TT	CC	CC	TT
CC	CC	CT	CT	AA	CT
CT	AA	CC	CT	AA	TT
CT	AC	CT	CT	AC	CC
CT	CC	CC	CT	AC	TT
CT	CC	TT	TT	AA	CC
TT	AC	CT	TT	AA	CT
TT	CC	CT	TT	AC	TT
TT	CC	TT			

Supplementary Figure S2: A. Overall survival curves according to the favorable and unfavorable genetic profiles obtained from the 3-way SDR analysis of the combinations of the *VEGFR-2 (rs11133360)* and $P2X_7R$ *(rs3751143, rs208294)* genotypes. Survival curves were calculated by the Kaplan Meier method. *CI, confidence interval*. **B.** Favorable and unfavorable genetic profiles from the genetic interaction analysis of *VEGFR-2 (rs11133360)* and $P2X_7R$ *(rs3751143, rs208294)* genotypes.

Supplementary Table S1: Multivariate Cox regression model

Variables	*HR*	*p*	*95%CI*
Age	0.94	0.018	0.88 – 0.99
ECOG PS	1.22	0.62	0.56 – 2.64
Gleason Score	5.9	0.003	1.79 – 19.56
Genetic profile			
Unfavorable	1	-	-
Favorable	0.31	0.003	0.15 – 0.66
MTS sites at the diagnosis	1.34	0.34	0.73 – 2.44
Therapy	0.88	0.73	0.43 – 1.8

MTS sites at the diagnosis and therapy were added to this model to further adjust even if not significant at univariate analysis (Table 1). ECOG PS, Eastern Cooperative Oncology Group performance status; HR, hazard ratio; CI, confidence interval; MTS, metastases; ECOG PS and MTS were analysed as continuous variables. Gleason Score represents the risk difference between patients with < 7 (reference) and ≥ 7. Therapy represents patients who received chemotherapy (none, 1 and ≥ 2 lines) and hormonal treatment.

Supplementary Table S2: Patient's characteristics of favorable and unfavorable genetic profile groups

	Favorable genetic profile	Unfavorable genetic profile	*P*
**Age**			
mean ± SD	69.9 ± 8.2	70.4 ± 8.97	
median (range)	70.5 (48 – 87)	72 (48 – 91)	0.789
**ECOG PS, *n* (%)**			
0	40 (76.92)	29 (67.44)	
1	11 (21.15)	11 (25.58)	
2	1 (1.92)	3 (6.98)	0.383
missing	1	1	
**MTS sites at the diagnosis, *n* (%)**			
0	35 (68.63)	16 (37.21)	
1	15 (29.41)	20 (46.51)	
2	1 (1.96)	7 (16.28)	*0.003*
missing	2	1	
**Gleason Score, *n* (%)**			
< 7	8 (22.22)	4 (12.90)	
≥ 7	28 (77.78)	27 (87.10)	0.321
missing	17	13	
**Therapy (chemo), *n* (%)**			
No	5 (9.43)	0	
1 line	15 (28.30)	9 (20.93)	
≥ 2 lines	33 (62.26)	34 (79.07)	0.063

ECOG PS, Eastern Cooperative Oncology Group performance status; MTS, metastases; Therapy represents patients who received chemotherapy (none, 1 and ≥ 2 lines). MTS sites at the diagnosis was the only variable statistically significant. This variable was used in multivariate COX regression to obtain an adjusted model. Simple *t*-test was used to compare continuous variables. Categorical variables were compared by the use of the $\chi 2$ test.
